# Nuclear Receptors in the Pathogenesis and Management of Inflammatory Bowel Disease

**DOI:** 10.1155/2019/2624941

**Published:** 2019-01-21

**Authors:** Longgui Ning, Xinhe Lou, Fenming Zhang, Guoqiang Xu

**Affiliations:** Department of Gastroenterology, First Affiliated Hospital, Zhejiang University School of Medicine, Hangzhou 310003, China

## Abstract

Nuclear receptors (NRs) are ligand-dependent transcription factors that regulate the transcription of target genes. Previous epidemiological and genetic studies have documented the association of NRs with the risk of inflammatory bowel disease (IBD). Although the mechanisms of action of NRs in IBD have not been fully established, accumulating evidence has demonstrated that NRs play complicated roles in regulating intestinal immunity, mucosal barriers, and intestinal flora. As one of the first-line medications for the treatment of IBD, 5-aminosalicylic acid (5-ASA) activates peroxisome proliferator-activated receptor gamma (PPAR*γ*) to attenuate colitis. The protective roles of rifaximin and rifampicin partly depend on promoting pregnane X receptor (PXR) expression. The aims of this review are to discuss the roles of several important NRs, such as PPAR*γ*, PXR, vitamin D receptor (VDR), farnesoid X receptor (FXR), and RAR-related orphan receptor gammat (ROR*γ*t), in the pathogenesis of IBD and management strategies based on targeting these receptors.

## 1. Introduction

Inflammatory bowel disease (IBD), including ulcerative colitis (UC) and Crohn's disease (CD), is a nonspecific chronic inflammatory disease that affects the gastrointestinal wall. In recent decades, the prevalence of IBD has increased globally. The highest reported prevalence rates are in Europe, with 505 cases of UC per 100,000 individuals in Norway and 322 cases of CD per 100,000 individuals in Germany [1, 2]. Clinically, IBD is characterized by relapsing symptoms, such as diarrhea, colorectal bleeding, and abdominal pain. In patients with CD, intestinal stricture and fistula formation are common and may require surgery. Furthermore, patients with long-term IBD are at increased risk of developing colitis-associated cancer [3]. However, the pathogenesis of IBD is not completely understood. Innate and adaptive immune cells and inflammatory molecules play crucial roles in the pathogenesis of IBD [4]. Tumor necrosis factor alpha (TNF-*α*) is one of the most important cytokines in IBD pathogenesis; antibodies against TNF-*α* are commonly prescribed therapeutics for IBD patients. However, nearly 40% of patients do not respond to anti-TNF-*α* treatment [5, 6]. Drugs targeting other pathways, such as anti-IL-23/IL-17 therapy and antiadhesion therapy, and small molecule inhibitors, like JAK inhibitors or S1P receptor modulators, appear promising for the management of IBD [7].

Nuclear receptors (NRs) are ligand-dependent transcription factors that activate or inhibit the transcription of their target genes. To this point, 48 NR family members have been found in human and 49 have been identified in mouse [8]. The NR family includes classic NRs and orphan receptors. Classic NRs recognize ligands like steroids, thyroid hormones, and vitamin metabolites. The basic structure of classic NRs consists of an N-terminal A/B domain, a DNA-binding domain, and a C-terminal ligand-binding domain [9]. Orphan receptors have similar structures to classical NRs, but their physiological ligands have initially been unclear; this class includes peroxisome proliferator-activated receptor gamma (PPAR*γ*), farnesoid X receptor (FXR), pregnane X receptor (PXR), RAR-related orphan receptor gammat (ROR*γ*t), and hepatocyte nuclear factor 4 alpha (HNF4*α*) [10]. When ligands bind their corresponding NRs, the NRs undergo conformational changes and recruit coactivators, leading to the dissociation of corepressors and subsequent transcription initiation [11].

In the gut, NRs play a broad range of intestinal functions, including nutrient absorption and transport, solute and water absorption/secretion, gut–liver communication, and gut microbiome regulation [12–14]. Moreover, several members of the NR family are involved in immune regulation [15]. As the gut immune system comprises 70%–80% of the body's immune cells, dysregulation of NR signaling may underlie the mechanisms of intestinal inflammatory diseases such as IBD. In this review, we provide a summary of the current research on the roles of several NRs in the pathogenesis of IBD and present novel insights into the management of IBD by targeting NRs.

## 2. NRs and IBD

### 2.1. PPAR*γ*

PPAR*γ*, also called NR1C3, belongs to the PPAR subfamily of NRs. PPAR*γ* is highly expressed in the adipose tissue and gut, and it regulates insulin resistance and adipogenesis [16, 17]. Clinically, PPAR*γ*-activating thiazolidinedione drugs like rosiglitazone or pioglitazone are used as antidiabetic drugs. Upon ligand binding, PPAR*γ* heterodimerizes with retinoid X receptor and regulates downstream gene transcription. In adipocytes, CD36, fatty acid-binding protein 4, adiponectin, and CCAAT/enhancer-binding protein are downstream targets of PPAR*γ*. PPAR*γ* also improves insulin resistance by promoting alternative macrophage activation, as demonstrated by the lower insulin sensitivity of macrophage-specific PPAR*γ* knockout (KO) mice compared to that of wild-type mice [18]. In mouse macrophages, ligand-dependent activation of PPAR*γ* leads to its SUMOylation, leading PPAR*γ* to interact with histone deacetylase 3 complexes at the promoters of inflammatory genes, thus repressing NF-*κ*B target gene transcription [19]. PPAR*γ* also exerts protective effects against lung inflammation and sepsis by regulating innate and adaptive immunity [20, 21]. Endogenous lipophilic species, including polyunsaturated fatty acids and eicosanoids, are natural ligands of PPAR*γ*.

Several studies have demonstrated reduced expression of PPAR*γ* in UC patients. Dubuquoy et al. observed lower expression of PPAR*γ*, which was confined to intestinal epithelial cells (IECs), in patients with UC than in patients with CD and healthy controls [22]. Although other reports have identified several PPAR*γ* genetic variants related to IBD susceptibility [23–25], they have not identified mutations in the PPAR*γ* gene in patients with UC; the differences in the findings may be associated with the ethnic differences between the study populations. Su et al. were the first to demonstrate that PPAR*γ* ligands, such as 15-deoxy-∆12,14 prostaglandin J2 (15d-PGJ2) and troglitazone, have anti-inflammatory effects in Caco-2 cells and mouse colitis models [26]. 15d-PGJ2 and troglitazone inhibit IL-8 and MCP-1 secretion in IL-1*β-*stimulated Caco-2 cells by preventing the activation of NF-*κ*B via an I*κ*B-*α*-dependent pathway. Numerous studies [27–31] have since assessed the anti-inflammatory effects of various PPAR*γ* ligands in different models of mouse colitis with gratifying results. In 2008, a randomized placebo-controlled trial demonstrated that administration of rosiglitazone improved clinical responses and the rate of clinical remission at week 12 compared with a placebo in patients with mild to moderate UC [32]. There were rare serious adverse events. Therefore, rosiglitazone appears to be efficacious and safe for the treatment of active UC.

Representative animal studies that examined the potential roles of NRs, including PPAR*γ*, in colitis are shown in Table 1. 5-Aminosalicylic acid (5-ASA) is a widely used first-line medication for the treatment of IBD, but the anti-inflammatory mechanism of 5-ASA remains unclear. Rousseaux et al. demonstrated that 5-ASA administration protects against colitis in wild-type, but not PPAR*γ*-heterozygous, mice [33]. Furthermore, they revealed that 5-ASA promotes PPAR*γ* translocation from the cytoplasm to the nucleus in IECs, thus regulating the transcription of downstream genes [33]. The protective effects of 5-ASA are dependent on PPAR*γ* expression in IECs, as confirmed in IEC-specific PPAR*γ* KO mice [34]. IEC-specific PPAR*γ* KO mice have increased susceptibility to dextran sodium sulfate- (DSS-) induced colitis. However, rosiglitazone may function through a PPAR*γ*-independent pathway to suppress IL-6, TNF-*α*, and IL-1*β* production, as rosiglitazone administration attenuates colitis in IEC-specific PPAR*γ* KO mice [34]. Later, several studies using macrophage- or CD4+ cell-specific PPAR*γ* KO mice revealed that the expression of PPAR*γ* in macrophages or CD4+ T cells protects against colitis [35–37]. Thus, PPAR*γ* expression in IECs and lamina propria mononuclear cells is protective against colitis. Further studies are needed to discover if the expression of PPAR*γ* in other immune cells, such as neutrophils or dendritic cells, has similar effects.

### 2.2. VDR

VDR (NR1I1) is the cellular receptor for 1,25-dihydroxyvitamin D (1,25[OH]_2_ vitamin D_3_), which has multiple regulatory effects on human metabolism, immunity, and cancer [38, 39]. The majority of vitamin D in the body is derived from photosynthesis in the skin driven by ultraviolet light irradiation, whereas a lesser part derives from dietary vitamin absorption in the small intestine. Vitamin D can be converted to the active hormone 1,25 (OH)_2_D_3_ via 25-hydroxylation in the liver and 1*α*-hydroxylation in the kidney [40]. However, 25 (OH) D, the circulating vitamin D metabolite, is measured to determine vitamin D levels.

A growing body of epidemiological studies has documented the association of vitamin D deficiency with an increased risk of IBD [41–43]. A meta-analysis summarized 4 polymorphisms (TaqI, BsmI, FokI, and ApaI) in the VDR gene that are associated with susceptibility to CD and UC [44]. Previous genome-wide association studies have identified several genetic variants that influence serum levels of vitamin D [45, 46]; the ability of those genetic variants to indirectly influence susceptibility to IBD remains to be investigated. Zator et al. suggested that low vitamin D levels may lead to earlier cessation of TNF-*α* therapy [47]. Low vitamin D levels in the plasma are associated with a poor prognosis, such as higher risk of surgery [48] or increased risk of clinical relapse, in patients with UC [49]. A more comprehensive analysis of vitamin D status in IBD is available in a recent review [40]. In a randomized double-blind placebo-controlled study, daily oral supplementation with 1200 IE vitamin D_3_ increased serum vitamin D levels and reduced the risk of relapse in CD patients from 29% to 13% (*P* = 0.06) [50]. However, as the result was not statistically significant, further studies with larger populations are needed. In another study, 300,000 IU intramuscular vitamin D decreased the serum erythrocyte sedimentation rate and high-sensitivity C-reactive protein levels in UC patients in remission after 90 days [51].

The mechanisms by which vitamin D exerts protective effects on IBD are complicated since vitamin D is widely recognized as a regulator of the immune system through its effects on T cells [52, 53], macrophages, and dendritic cells [54, 55]. Vitamin D dietary deficiency exacerbates the symptoms of enterocolitis in IL-10 KO mice, whereas dietary vitamin D supplementation improves diarrhea and prevents weight loss [56]. In a cell transfer model of enteritis, CD4+ CD45RB^high^ T cells from VDR KO mice induced more severe colitis in recombinase-activated gene (Rag) 2 KO recipient mice than CD4+ CD45RB^high^ T cells from wild-type mice [57].

VDR expression in IECs is also protective against colitis. In Caco-2 cell cultures, vitamin D enhances intestinal integrity as evidenced by higher expression of tight junction proteins and transepithelial electrical resistance, whereas VDR knockdown destroys intestinal integrity [58]. IEC-specific VDR KO mice display worse colitis and higher expression of TNF-*α*, IL-1*β*, and MCP-1 than wild-type mice [59]. Interestingly, epithelial-specific human VDR transgenic mice are protected from developing colitis due to the preservation of the mucosal barrier and protection of IECs from apoptosis through blocking TNF-*α*-induced p65 binding to the *κ*B site of the PUMA gene promoter [60]. VDR also plays protective roles in colitis by regulating the intestinal microbiota; the absence of intestinal epithelial VDR leads to defective autophagy and affects microbial assemblage [61]. In 2 recent studies, the microbial communities of patients with CD and UC changed dramatically after early vitamin D administration compared with those in healthy controls [62, 63]. Thus, vitamin D administration may be an effective supplementary treatment for IBD.

### 2.3. PXR

PXR (NR1I2) is an NR that mainly participates in the regulation of genes involved in drug transport and metabolism [64]. Human PXR is highly expressed in the small intestine, colon, and liver, with lower expression in the stomach [65]. It has a broad range of ligands, ranging from exogenous prescription drugs and dietary supplements to endogenous hormones and bile acids [64]. Although the ligand-binding domains in human and mouse PXR share approximately 80% amino acid similarity, the agonistic effects stimulated by their ligands differ. For example, pregnenolone-16-carbonitrile (PCN) is a rodent-specific PXR agonist, whereas rifaximin and rifampicin are human PXR agonists.

Many studies have investigated the genetic associations of the PXR gene with IBD with inconclusive results. Dring et al. found that SNPs −23585, 24381, and 8055 in PXR are statistically associated with IBD in an Irish cohort [66]. In a Spanish population, patients with extensive UC were more likely to carry the −25385T allele than individuals with left-sided colitis and healthy subjects [67]. In a Caucasian cohort, several rare PXR/NR1I2 haplotypes were highly associated with CD susceptibility [68]. However, a meta-analysis that included 6 studies suggested that 3 PXR SNPs (rs1523127, rs2276707, and rs6785049) had no obvious influence on the risk of IBD in Caucasians [69]. Notably, the number of original studies covered by the meta-analysis was limited, and further studies with various populations are needed to confirm the results.

Several randomized double-blind placebo-controlled studies have demonstrated that rifaximin administration to active CD patients results in a higher 12-week clinical remission rate than a placebo (Table 2) [70, 71]. Rifaximin also effectively maintains remission in CD patients who had achieved remission with standard therapy (100% of rifaximin-treated versus 87% of placebo-treated patients) [72]. However, the efficacy of rifaximin in patients with UC is less well understood.

Previous studies revealed the expression of PXR in human CD4+ and CD8+ T lymphocytes, CD19+ B lymphocytes, and CD14+ monocytes, but not in bone marrow-derived mouse macrophages [73]. In PXR-deficient mice, T lymphocytes undergo excessive proliferation and exhibit higher CD25 expression than in wild-type mice. PXR activation in both mouse and human T cells inhibits T cell proliferation and CD25 and IFN-*γ* expression in vitro. PXR activation by PCN is protective against DSS-induced colitis due to the activation of phase II enzymes and cellular efflux transporters, such as GSTa1, MDR1a, and MRP2, which alleviates the expression of the proinflammatory cytokines IL-6, TNF-*α*, ΜCP-1, and IL-1*β* [74]. However, in PXR KO mice, the protective effects of PCN are abolished. Mechanistically, PXR activation inhibits the activating effects of TNF-*α* on proinflammatory NF-*κ*B [74]. Rifampicin is a synthetic agonist for human, but not rodent, PXR. Using primary fetal human colon epithelial cells, Mencarelli et al. revealed that rifampicin suppresses the expression of IL-6, TNF-*α*, and IL-8 and promotes the expression of TGF-*β* by repressing lipopolysaccharide- (LPS-) induced NF-*κ*B DNA-binding activity, whereas PXR silencing completely abrogates the protective effects of rifaximin [75]. Stimulation of Caco-2 cells with PXR agonists, such as rifaximin, rifampicin, and SR12813, promotes wound closure and intestinal barrier repair. These effects are dependent on p38 MAPK-mediated cell migration, with no effects on cell proliferation [76]. As an antibiotic, rifaximin inhibits bacterial translocation, adhesion, and internalization [77, 78]. Several other publications have confirmed the anti-inflammatory and barrier-preserving effects of PXR agonists [79–81].

### 2.4. FXR

FXR (NR1H1) is an NR involved in many aspects of human physiology, including development, reproduction, and metabolism [82]. Similar to other NRs, the structure of FXR has been well characterized and consists of a DNA-binding domain in the N-terminal region and a ligand-binding domain in the C-terminal region. The most important function of FXR is the regulation of bile acid homeostasis, as reviewed in other publications [83, 84]. Interestingly, bile acids like chenodeoxycholic acid (CDCA) are endogenous ligands for this NR, so FXR is also known as a bile acid NR. Synthetic molecules, such as GW4064, fexaramine, or AGN34, and semisynthetic agonists, such as 6-ECDCA or INT-747, are powerful activators of FXR signaling [85].

Attinkara et al. [86] studied the association of 5 NR1H4 gene variants (rs3863377, rs7138843, rs56163822, rs35724, and rs10860603) with IBD. They observed that the NR1H4 SNP rs3863377 appears less frequently in IBD cases than in non-IBD controls, whereas the variant rs56163822 is less prevalent in non-IBD controls [86]. However, these genetic associations could not be demonstrated in Dutch IBD patients [87].

Although mRNA levels of FXR do not differ between patients with IBD and healthy controls, the expression of small heterodimer partner in the ileum is lower in patients with CD than in healthy controls, indicating reduced FXR activity in CD [87]. Using FXR KO mice, researchers demonstrated that FXR is expressed by immune cells and exerts regulatory effects, mainly on innate immune cells [88]. Exposure of LPS-stimulated macrophages to INT-747, a synthetic FXR ligand, represses the expression of the proinflammatory factors IL-6, TNF-*α*, IFN-*γ*, and IL-1*β* and induces the expression of SHP [88]. FXR activation by INT-747 stabilizes the nuclear corepressor NCoR on the NF-*κ*B-responsive element within the IL-1*β* and iNOS promoters. In addition to its effects on acute colitis, INT-747 protects against the development of chronic intestinal inflammation and fibrosis formation [88]. FXR activation by obeticholic acid (OCA) is associated with the retention of dendritic cells in the spleen, but not in mesenteric lymph nodes, thereby alleviating inflammatory cell infiltration of the colon [89]. It will be interesting to investigate if the modulation of FXR in the adaptive immune system is beneficial for intestinal inflammation.

In addition to its regulatory effects on the innate immune system, FXR protects the epithelial barrier. FXR activation in the intestines of wild-type mice and in enterocytes downregulates the expression of the proinflammatory cytokines IL-6, MCP-1, and IL-1*β* and preserves epithelial barrier integrity [90]. In ex vivo experiments, INT-747 significantly downregulates TNF-*α*, IL-17, and IFN-*γ* production in activated human peripheral blood mononuclear cells, purified CD14+ monocytes, and dendritic cells, and in the lamina propria mononuclear cells of patients with IBD. On the other hand, deoxycholic acid and GW4064 significantly inhibit wound closure in epithelial monolayers by inducing the nuclear accumulation of FXR [91], whereas ursodeoxycholic acid (UDCA) promotes wound healing. As UDCA functions as a very weak FXR agonist [92], its effects may be mediated through FXR-independent mechanisms. However, high concentrations of bile acids induce fluid and electrolyte secretion in the colon [93], leading to diarrhea in some patients. Mroz et al. discovered that FXR activation by GW4064 inhibits fluid and electrolyte secretion in an ovalbumin-induced allergic diarrhea model and a cholera toxin-induced intestinal fluid accumulation model [94]. At the molecular level, FXR activation attenuates apical Cl (-) currents by inhibiting the expression of cystic fibrosis transmembrane conductance regulator channels and basolateral Na^+^/K^+^-ATPase transport.

### 2.5. ROR*γ*t

The ROR subfamily of NRs consists of 3 members: ROR*α*, ROR*β*, and ROR*γ* [95]. ROR*γ* and ROR*γ*t are the 2 isoforms transcribed from the RORC locus. ROR*γ*t is selectively expressed in immune organs, including the thymus. It acts as a critical transcription factor for Th17 cell differentiation and plays an important role in Th17-related chronic inflammation and autoimmune diseases [96]. Until now, no endogenous ligands for ROR*γ*t have been identified. However, multiple groups are working to identify small molecule inhibitors for ROR*γ*t that bind its ligand-binding domain [97].

Data from IBD patients and mouse models of colitis have revealed that T cells, especially the Th1–Th17 and Th17-Treg axes, play important roles in the regulation of intestinal immunity [98]. Adoptive transfer of IL-17A-, IL-17F-, or IL-22-deficient T lymphocytes into RAG1-null mice results in more severe colitis than that caused by wild-type T cells [99]. In contrast, transfer of ROR*γ*t-deficient T cells into RAG1-null mice fails to augment IL-17 expression and does not cause colitis [99], indicating a crucial role for ROR*γ*t-expressing Th17 cells in colitis. Although previous clinical trials in patients with rheumatoid arthritis showed a benefit of blocking IL-17A with secukinumab, a phase 2 clinical trial that enrolled patients with active CD demonstrated that IL-17A blockade is ineffective and leads to higher rates of adverse events than treatment with a placebo [100]. These findings suggest a protective role of IL-17A in CD. A later study revealed that IL-17A had a redundant but highly pathogenic role in gut inflammation [99]. Therefore, developing small molecule inhibitors for ROR*γ*t, rather than for IL-17A, may be an alternative approach to control Th17 immunity in IBD.

The Littman group identified digoxin, a cardiac glycoside used in heart failure patients, as a specific inhibitor of ROR*γ*t transcriptional activity [101]. Furthermore, they demonstrated that digoxin inhibits Th17 cell differentiation in the mouse experimental autoimmune encephalomyelitis model without influencing other T cell populations. Similar to the results of previous studies, digoxin efficiently attenuated the colitis induced by adoptive transfer of CD45RB+ CD4 T cells by downregulating Th17 cytokines and receptors, such as IL-17A, IFN-*γ*, and IL-23R, but did not influence mucosal TNF-*α* expression [102]. It will be important to investigate if digoxin holds therapeutic value for CD patients who are unresponsive to anti-TNF-*α* therapy.

GSK805, an oral inhibitor of ROR*γ*t, suppresses intestinal inflammation by eliminating Th17 cells and preserving group 3 innate lymphoid cells in IL-10 KO- and *Citrobacter rodentium*-induced colitis models [103]. Using a fluorescence resonance energy transfer assay, Xiao et al. identified TMP778 and TMP920 as highly potent and selective ROR*γ*t inhibitors [104]. Ursolic acid was also identified in a compound library screen as an inhibitor of ROR*γ*t [105]. The therapeutic roles of these new small molecule antagonists in the treatment of IBD remain to be elucidated.

### 2.6. Other NRs

Many other NRs play important roles in IBD in addition to those mentioned above. However, the research literature on those NRs in IBD is relatively limited. We briefly summarized the roles in IBD of several other NRs (Table 2), including Nur77 (NR4A1) [106, 107], liver receptor homolog 1 (LRH-1/NR5A2) [108, 109], liver X receptor (LXR/NR1H) [110], constitutive androstane receptor (CAR/NR1I3) [111], hepatocyte nuclear factor-4 alpha (HNF4*α*/NR2A1) [112–114], and NR2F6 [115].

## 3. Crosstalk between NRs and Gut Microbiota in IBD

Distinct fecal microbial communities were found in patients with IBD and healthy subjects [116–118]. The microbiomes of patients with IBD were characterized by lower abundances of Bacteroidetes, Firmicutes, and *Faecalibacterium prausnitzii* [119]. Recently, fecal microbiota transplantation has been shown to induce remission in patients with active UC with no obvious adverse events [120, 121]. As NRs have a broad range of functions and are highly expressed in the gut, the interplay between NRs and the gut microbiota remains a highly researched topic. We have summarized the recent advances in the understanding of the interactions between VDR, PPAR*γ*, FXR, and gut microbiota.

Data from human and animal studies suggest that vitamin D supplementation changes the gut microbiome in patients with IBD by increasing the abundance of potentially beneficial bacterial strains. In patients with active UC, cholecalciferol administration for 8 weeks led to lower fecal calprotectin levels and an increase in the abundance of Enterobacteriaceae, with no change in the overall microbial diversity [63]. However, no changes in Enterobacteriaceae abundance were observed in patients with inactive UC and non-IBD controls who received the same dose of cholecalciferol [63]. Similarly, early vitamin D administration to patients with CD for 1 week increased the abundance of several species, such as *Alistipes*, *Barnesiella*, *Roseburia*, and *Anaerotruncus* [62]. Mice that are fed with a vitamin D-deficient diet are predisposed to more severe colitis and elevated levels of bacteria in colonic tissue than mice fed with a vitamin D-replete diet [122]. The expression of angiogenin 4, an antimicrobial protein, is lower in vitamin D-deficient mice than in wild-type mice [122]. Cyp27b1 is an enzyme that catalyzes the precursor 25-hydroxycholecalciferol into the active form of vitamin D, 1,25 (OH)_2_D_3_. Cyp27b1 KO mice are susceptible to DSS-induced colitis and have more bacteria from the Bacteroidetes and Proteobacteria phyla and fewer bacteria from Firmicutes and Deferribacteres in their feces [123]. Interestingly, 1,25 (OH)_2_D_3_ supplementation improves colitis and decreases the abundance of Helicobacteraceae. In intestinal epithelial-specific VDR KO (VDR^∆IEC^) mice models compared to wild-type mice, lower expression of ATG16L1 and lysozyme and impaired antimicrobial Paneth cell function have been observed [61]. VDR^∆IEC^ mice have higher abundances of *E. coli* and Bacteroides and lower abundances of butyrate-producing bacteria than wild-type mice. The bacterial product butyrate upregulates VDR in HCT116 cells and reverses impaired autophagy [61]. Moreover, lower expression of intestinal epithelial VDR correlates with reduced ATG16L1 expression and a higher abundance of intestinal *Bacteroides fragilis* in UC patients compared to healthy controls [61]. In a mouse model of *Salmonella* infection, *Salmonella* inhibits the expression, distribution, transcriptional activity, and target gene expression of VDR, resulting in elevated NF-*κ*B activity in the mucosa and increased susceptibility to colitis [124].

PPAR*γ* is another NR that mediates host–microbiota crosstalk in IBD. During postembryonic development of the gut, *Enterococcus faecalis* from newborn babies promotes PPAR*γ* transcriptional activity through the phosphorylation of PPAR*γ* [125]. Phosphorylated PPAR*γ* transcriptionally activates IL-10 to modulate innate immune function. Supplementation with the probiotic *Lactobacillus crispatus* M247 in vivo and in vitro increases PPAR*γ* levels and transcriptional activity [126]. Further studies revealed that *L. crispatus* M247-derived H_2_O_2_ that is responsible for the activation of PPAR*γ* as the transcriptional activity of PPAR*γ* is negated by antioxidants or an H_2_O_2_ scavenger [126]. A later study analyzed the bacteria–host interactions of 57 commensal bacterial strains, with a focus on PPAR*γ* transcriptional activity [127]. They observed that PPAR*γ* transcriptional activity was activated by ERK1/2 in the presence of butyrate and propionate in the conditioned media from anaerobic cultures. The microbiota-derived metabolite butyrate prevents colitis by inhibiting histone deacetylase 1 and regulating innate and adaptive immunity [128, 129]. Depletion of butyrate-producing microbes by antibiotic treatment leads to reduced PPAR*γ* expression in colonocytes and dysbiotic Enterobacteriaceae expansion [130]. On the other hand, the microbial dysbiosis in the murine small intestine induced by a high-fat diet can be ameliorated to the standard composition by administration of the PPAR*γ* activator rosiglitazone. The ability of PPAR*γ* activation to reverse microbial dysbiosis in patients with IBD will be an important line of future research.

Recent studies revealed that FXR activation by fexaramine shapes the gut microbiota to activate TGR5/GLP-1 signaling, which improves metabolism [131]. Healthy volunteers given OCA had lower endogenous bile acid levels and a reversible induction of Gram-positive bacteria in their small intestines, which is consistent with the effects observed in mice that were treated with OCA [132]. In addition, metformin alters the gut microbiota composition in humans [133]. Metagenomic and metabolomic analyses in patients with type 2 diabetes revealed lower levels of *B. fragilis* and higher levels of glycoursodeoxycholic acid (GUDCA) in the gut following metformin supplementation [134]. When mice fed with a high-fat diet and metformin are colonized with *B. fragilis*, the metabolic improvements derived from metformin treatment are reversed. GUDCA was later identified as an intestinal FXR antagonist that improves metabolic symptoms in mice with established obesity [134]. Therefore, metformin acts partly via a *B. fragilis*/GUDCA/intestinal FXR axis to improve metabolic dysfunction.

## 4. Conclusion and Perspectives

**Figure 1:**
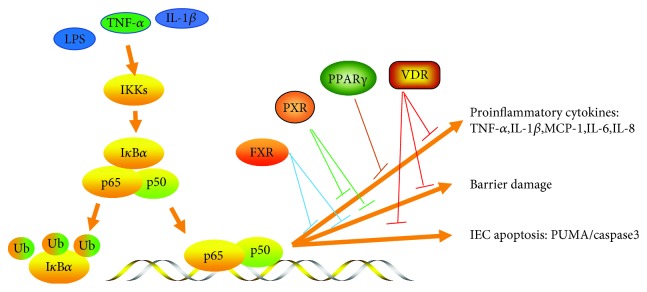
NRs and cytokines signaling. Various stimulus, such as LPS, TNF-*α*, and IL-1*β*, activate NF-*κ*B signaling pathway and p65-p50 binds to the promoter of downstream genes in immune cells or IECs, leading to proinflammatory cytokines production, barrier damage, and IEC apoptosis. NRs blocked the binding of the p65-p50 complex to the promoter and improved the damage effects induced by various stimulus.

In this review, we provide a comprehensive overview of NRs in IBD, which covers genetic associations, animal models, clinical trials, and crosstalk with gut microbiota. Activation of NRs, such as PPAR*γ*, VDR, FXR, and PXR, alleviates colitis by restraining NF-*κ*B-mediated proinflammatory cytokines production and inhibiting inflammatory cytokine-induced IEC apoptosis and intestinal barrier damage (Figure 1). Although considerable evidence has confirmed the crosstalk between NRs and the gut microbiota, the roles of FXR and PPAR*γ* activation in shaping the gut microbiota in IBD patients have not been investigated. We encourage research in this area to facilitate the development of novel strategies for the treatment of IBD based on the restoration of healthy host–microbiome interactions by targeting NRs. Furthermore, an in-depth understanding of the crosstalk between NRs and the gut microbiota may provide novel insights into other gastrointestinal diseases that are associated with the gut microbiota, like colorectal cancer and irritable bowel syndrome.

NR dysregulation can alter the intestinal microbiota, damage the intestinal barrier, and imbalance intestinal immunity, thereby contributing to the pathogenesis of IBD. As such, targeting NRs with their ligands or inhibitors may constitute a novel therapeutic approach for the management of IBD. Most of the ligands or inhibitors are small molecules, which could be given orally and would not elicit immunogenic side effects. These drugs also have lower manufacturing costs than antibody-based therapies. A summary of previous randomized placebo-controlled trials on NR agonists for the treatment of IBD is shown in Table 3. However, despite the benefits of the NRs in those clinical trials, NR-targeting drugs may not be sufficient as monotherapies given the complicated pathogenesis of IBD. It will be important to consider if NRs can be used in adjuvant therapies together with currently available drugs.

Due to the multiple regulatory effects of NRs in the human body, care should be taken when applying NR modulators as therapeutic drugs for clinical use. For example, although PXR activation is protective against colitis, it promotes FGF19-dependent colon cancer aggressiveness in humans and mice [135]. Given the possible tumor-promoting effects of PXR, caution should be exercised in the use of PXR activators to treat IBD. These dual effects highlight the need for additional research to provide a better understanding of the long-term safety of NR-targeted therapy for IBD. In addition, NRs such as PPAR*γ* and FXR are involved in liver energy metabolism and bile acids homeostasis [136], so their activation may influence normal liver function. Thus, developing gut-specific NR modulators will be an important direction of future research.

## Figures and Tables

**Table 1:** Representative animal studies examining the potential roles of NRs in colitis.

NRs	Study type	Functions and effects	Ref.
PPAR*γ*	Agonist: 5-ASA PPAR*γ* KO mice	PPAR*γ* is a target of 5-ASA underlying anti-inflammatory effects	[33]
	Agonist: rosiglitazone IEC-specific PPAR*γ* KO mice	PPAR*γ* expressed in the IEC has an endogenous role in protection against colitis	[34]
	CD4+ T cell-specific PPAR*γ* KO mice	PPAR*γ* in T cells is involved in preventing gut inflammation by regulating adhesion molecules and inflammatory mediators	[37]
	Agonist: pioglitazone Macrophage-specific PPAR*γ* KO mice	Macrophage-specific PPAR*γ* KO exacerbated colitis, impaired Treg compartment, and increased LP CD8+ T cells	[35]

VDR	Agonist: 1,25(OH)_2_ D-3 VDR KO mice	VDR preserves the integrity of junction complexes and the healing of the IEC	[58]
	Agonist: 1,25(OH)_2_ D-3 hVDR Tg and VDR KO mice	VDR signaling attenuates PUMA induction in IECs by blocking NF-*κ*B activation, leading to a reduction in IEC apoptosis	[60]
	IEC-specific VDR KO mice	Absence of intestinal epithelial VDR affects microbial assemblage and autophagy	[61]

PXR	Agonist: pregnenolone-16alpha-carbonitrile PXR KO mice	PXR agonist decrease mRNA expression of several NF-*κ*B target genes in a PXR-dependent manner	[74]
	Agonist: rifaximin, rifampicin SR12813, and PCN	Agonists enhanced intestinal epithelial repair by p38 MAP kinase-dependent way	[76]
	Agonist: rifaximin and SR12813	PXR regulates the IEC barrier by modulating cytokine-induced MLCK expression and JNK1/2 activation	[81]

FXR	Agonist: 6E-CDCA, INT-747 FXR KO mice	Colitis was exacerbated in FXR KO mice. FXR activation stabilizes corepressor NCoR on the NF-*κ*B responsive element	[88]
	Agonist: INT-747 FXR KO mice	FXR downregulates the expression of key proinflammatory cytokines and preserves epithelial barrier function	[90]
	Agonist: GW4064	FXR activation attenuated apical Cl (-) currents by inhibiting the expression of CFTR and Na (+)/K (+)-ATPase activity	[94]

ROR*γ*t	Inhibitor: digoxin	Digoxin downregulated Th17 cytokines	[102]
	Inhibitor: GSK805	GSK805 provided therapeutic benefit in intestinal inflammation and reduced the frequency of Th17 cells but not ILCs	[103]

NRs, nuclear receptors; PPAR*γ*, proliferator-activated receptor-*γ*; IEC, intestinal epithelial cells; KO, knockout; LP, lamina propria; VDR, vitamin D receptor; PXR, pregnane X receptor; FXR, farnesoid X receptor; ROR*γ*t, retinoid-related orphan receptor gammat; ILCs, innate lymphoid cells.

**Table 2:** Randomized placebo-controlled trials of NRs agonists in IBD.

NRs	Agonist	Design	Outcome	Ref.
PPAR*γ*	Rosiglitazone	Mild to moderately active UC rosiglitazone (*n* = 52) vs. placebo (*n* = 53) 4 mg twice daily vs. placebo	12W clinical response 44% of rosiglitazone vs. 23% of placebo	[32]

VDR	Vitamin D3	CD in remission 1200 IU vitamin D3 (*n* = 46) vs. placebo (*n* = 48) once daily	12M relapse rate: vitamin D3 13% vs. placebo 29%	[50]
	Vitamin D3	UC in remission 300,000 IU intramuscular vitamin D3 vs. 1 mL normal saline as placebo (*n* = 90)	90 days after intervention Vitamin D3 decreases ESR and hs-CRP levels and increase in LL37 gene expression	[51]

PXR	Rifaximin	Mild-to-moderate CD rifaximin 800 mg o.d.+ placebo o.d. (*n* = 25) rifaximin 800 mg b.d. (*n* = 29) placebo b.d. (*n* = 29)	12W clinical remission rate: 32%, 52%, 33% 12W clinical response rate: 48%, 67%, 41%	[70]
	Rifaximin	Moderately active CD rifaximin 800 mg (*n* = 98) vs placebo (*n* = 101)	12W remission rate: 62% of rifaximin vs. 43% of placebo	[71]
	Rifaximin	Moderately active CD in remission 800 mg of rifaximin (*n* = 83) b.d. vs. 800 mg placebo (*n* = 83)	12W remission rate: 100% of rifaximin vs. 87% of placebo	[72]

NRs, nuclear receptors; IBD, inflammatory bowel disease; PPAR*γ*, proliferator-activated receptor-*γ*; UC, ulcerative colitis; CD, Crohn's disease; W, week; VDR, vitamin D receptor; M, month; hs-CRP, high-sensitive C-reactive protein; ESR, erythrocyte sedimentation rate; PXR, pregnane X receptor.

**Table 3:** Brief summary of other NRs in IBD.

NRs	Study type	Functions and effects	Ref.
Nur77	Nur77 KO mice Agonist: cytosporone B	Nur Nur77 acts as a negative regulator of NF-*κ*B by directly interacting with TRAF6	[106]
	Nur77 KO mice	Nur77 inhibits inflammatory status of both macrophages and gut epithelial cells	[107]

LRH-1	LRH-1 heterozygous mice Epithelium-specific LRH-1-deficient mice	LRH-1 regulates intestinal immunity by mediating glucocorticoid synthesis in enterocytes	[108]
	Murine organoids Epithelium-specific LRH-1-deficient mice	LRH-1 maintains epithelial integrity and viability; prevents crypt death and injury	[109]

LXR	LXR KO mice Agonist: GW3965	LXR accelerates weight recovery and inhibits inflammatory mediators production	[110]

CAR	Agonist: TCPOBOP CAR KO mice	CAR promotes intestinal epithelial migration and wound healing	[111]

HNF4*α*	Intestine-specific HNF4*α*KO mice	HNF4*α* preserves mucin barrier and increases intestinal permeability	[112, 113]
	HNF4*α* knockdown	HNF4*α* modifies oxidative stress, inflammation, and lipoprotein assembly	[114]

NR2F6	NR2F6 KO mice	NR2F6 binds to the Muc2 promoter and transactivates Muc2 expression, alters intestinal permeability, and protects against colitis	[115]

NRs, nuclear receptors; KO, knockout; LRH-1, liver receptor homolog-1; CAR, constitutive androstane receptor; LXR, liver X receptor; HNF4a, hepatocyte nuclear factor 4alpha.
